# Serum Lipid Levels and Treatment Outcomes in Women Undergoing Assisted Reproduction: A Retrospective Cohort Study

**DOI:** 10.3389/fendo.2021.633766

**Published:** 2021-03-08

**Authors:** Wang-Yu Cai, Xi Luo, Erxidi Chen, Houyi Lv, Kaiyou Fu, Xiao-Ke Wu, Jian Xu

**Affiliations:** ^1^Fourth Affiliated Hospital, Zhejiang University School of Medicine, Yiwu, China; ^2^Department of Gynecology, Second Affiliated Hospital of Zhejiang Chinese Medical University, Hangzhou, China; ^3^Women’s Hospital, Zhejiang University School of Medicine, Hangzhou, China; ^4^Department of Obstetrics and Gynecology, First Affiliated Hospital, Heilongjiang University of Chinese Medicine, Harbin, China; ^5^Heilongjiang Province Hospital, Harbin, China

**Keywords:** lipid, reproduction, assisted reproduction, live birth, pregnancy, miscarriage

## Abstract

**Objective:**

To evaluate associations between serum lipid levels and treatment outcomes in women undergoing assisted reproduction.

**Materials and Methods:**

The study included 2011 women who underwent *in vitro* fertilization/intracytoplasmic sperm injection with fresh embryo transfer. Serum lipid evaluation included total cholesterol (TC), low-density lipoprotein cholesterol (LDL-C), high-density lipoprotein cholesterol (HDL-C), and triglycerides (TG). Ovarian stimulation outcomes included endometrial thickness and the number of oocytes retrieved, and reproductive outcomes included live birth, clinical pregnancy, and miscarriage.

**Results:**

Higher HDL-C quartiles were associated with more oocytes retrieved. Lower TC (quartile 1 odds ratio [OR] 1.59 [1.21–2.08], quartile 3 OR 1.36 [1.04–1.77]), LDL-C (quartile 1 OR 1.41 [1.07–1.86]), and TG (quartile 2 OR 1.39 [1.06–1.84]) were independently associated with clinical pregnancy after adjusting for potential confounders. Lower LDL-C (quartile 1 OR 2.22 [1.58–3.13], quartile 2 OR 1.78 [1.27–2.50], quartile 3 OR 1.51 [1.07–2.13]), TC (quartile 1 OR 1.39 [1.00–1.93]), TG (quartile 1 OR 1.44 [1.03–2.03], quartile 2 OR 1.46 [1.04–2.04], quartile 3 OR 1.44 [1.04–1.99]), and higher HDL-C (quartile 2 OR 0.71 [0.51–0.99]) were independently associated with live birth. Higher LDL-C (quartile 1 OR 0.44 [0.30–0.66], quartile 2 OR 0.49 [0.33–0.73], quartile 3 OR 0.63 [0.43–0.94]) and lower HDL-C (quartile 1 OR 1.60 [1.07–2.39]) were independently associated with miscarriage.

**Conclusions:**

Serum lipid levels were associated with treatment outcomes in women undergoing assisted reproduction.

## Introduction

Infertility, defined as the inability to conceive a child after 1 year, is estimated to affect millions of couples worldwide (1). Infertility and assisted reproduction technology are associated with considerable emotional and financial burdens, and assisted reproduction does not have a good success rate (2–4). In conjunction with the worldwide obesity epidemic (5), lipid abnormalities are common (6, 7). The success of assisted reproduction may be affected by the patient’s social, psychological, and physical status. Routine serum lipid screening includes total cholesterol (TC), low-density lipoprotein cholesterol (LDL-C), high-density lipoprotein cholesterol (HDL-C), and triglycerides (TG). Lipid disorders are hypothesized to play a role in female reproduction. The synthesis of steroid hormones in reproductive tissues occurs in thecal and granulosa cells, and utilizes cholesterol as the substrate for steroidogenesis (8). HDL-C and LDL-C both play important roles in the transport of cholesterol to ovarian tissue (9). Altered endometrial lipid levels may impair endometrial receptivity and early embryo implantation  (10). Acute atherosis and diffuse lipid infiltration can also be found in the placenta, and are pathognomonic signs of potential placental malfunction (11). These observations suggest possible links between serum lipids and reproductive health in women.

Some studies have reported associations between serum lipids and reproductive outcomes in subfertile women. In one prospective cohort study that included couples attempting pregnancy, increased serum free cholesterol concentrations in both men and women led to reduced fecundity (12). In couples with prior pregnancy loss, higher serum TC and TG were associated with less spontaneous pregnancy (13). Another study in women undergoing *in vitro* fertilization (IVF) suggests that HDL within the follicular fluid may play protective roles in the health of the human oocyte by reducing oocyte fragmentation (14, 15). It has also been reported that follicular fluid HDL had an antioxidative function and was associated with normal oocyte fertilization (16, 17).

Few studies have investigated associations between serum lipid profiles and treatment outcomes in women undergoing assisted reproduction, particularly after ruling out the effects of potential confounders such as body mass index (BMI). At 40%–50% the prevalence of lipid abnormalities is high (6, 7), and understanding associations between serum lipid levels and reproductive outcomes after assisted reproduction may provide an early, modifiable, and inexpensive treatment choice. The objective of the current study was to investigate associations between treatment outcomes and preconception serum TC, LDL-C, HDL-C, and TG levels in a cohort of women undergoing IVF/intracytoplasmic sperm injection (ICSI) cycles.

## Materials and Methods

### Study Design

The present retrospective study was conducted from January 2018 to October 2020 at a reproductive medical center of a university-affiliated hospital in mainland China. The study was approved by the ethics committee of the hospital (IRB-20200235-R), and was conducted in accordance with the Declaration of Helsinki. Because it was a retrospective study and only de-identified data were analyzed, the ethics committee waived the standard requirement for informed consent from the women in the study.

### Study Population

**Figure 1:**
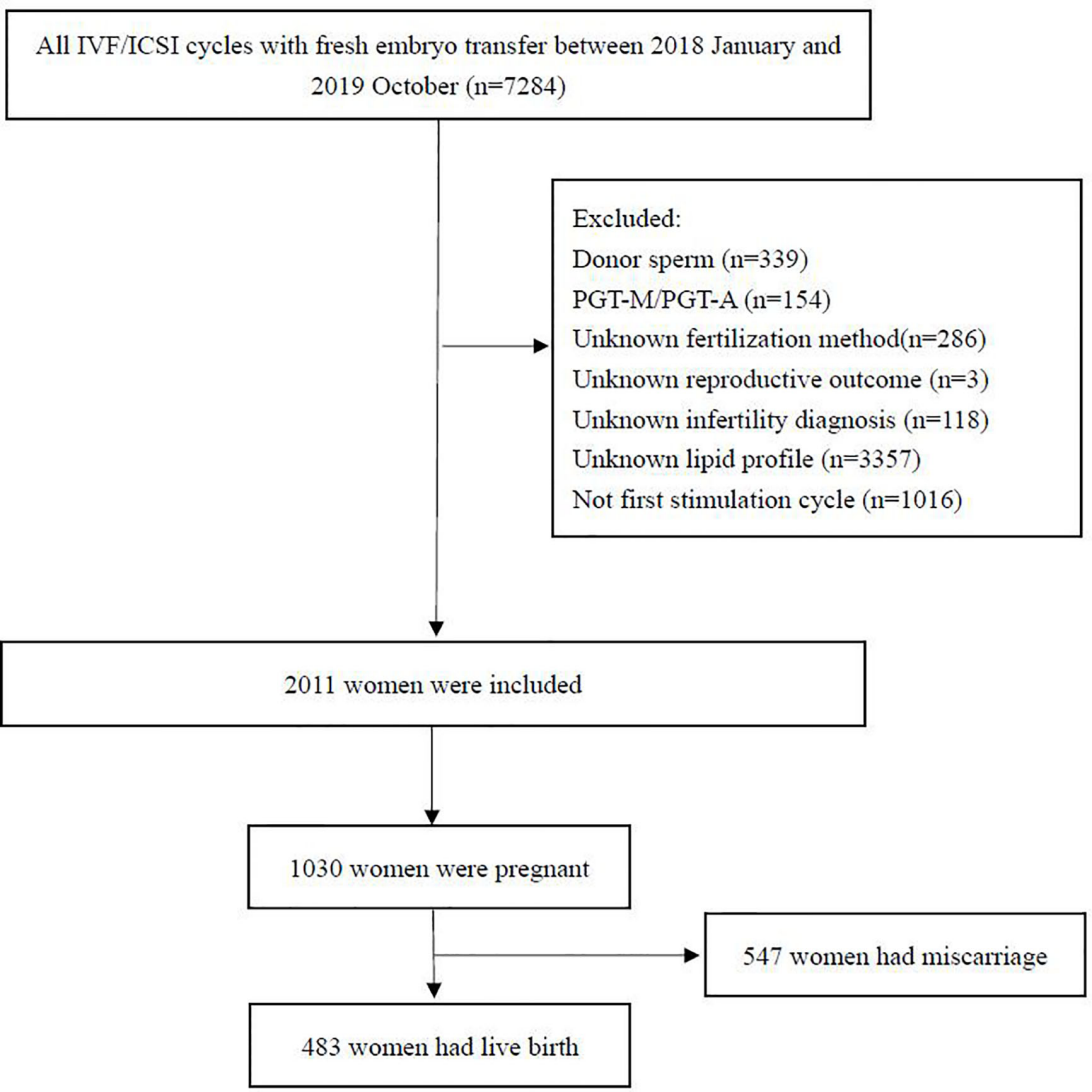
Flowchart for selecting women included in this study.

Patients aged between 18 and 45 years who underwent their first IVF/ICSI cycle with fresh embryo transfer from January 2018 to October 2020 were included in the study. Patients with infertility of any cause were included. The inclusion criteria were (1) IVF/ICSI with fresh embryo transfer; and (2) serum lipid levels evaluated before treatment. The exclusion criteria were (1) the use of an egg or sperm donor; (2) preimplantation genetic diagnosis or screening; (3) lack of serum lipid evaluation; (4) lack of treatment outcome data; and (5) incorrect information or important data missing from the database. Data from a total of 7,284 women were initially screened, of which 2011 were included in the final analysis. A flow chart representing the study procedure is shown in **Figure 1**.

During the initial evaluation age, height, weight, and current smoker status were recorded, and BMI was calculated. Reproductive characteristics included infertility diagnosis, primary infertility, and duration of infertility. Anti-Mullerian hormone (AMH), day 3 follicle-stimulating hormone (FSH), basal estradiol (E2), TC, LDL-C, HDL-C, TG, and glucose were measured. After treatment, ovarian stimulation days, total gonadotropin dose, E2 on the day of human choriogonadotropin (hCG) administration, endometrial thickness, fertilization procedure, number of oocytes retrieved, number of two pronuclei embryos, and number of embryo transfers were recorded.

### Laboratory Assessment

Preconception serum samples were collected on days 2–4 of the women’s menstrual cycles. TG, LDL-C, HDL-C, and TG were measured using a Beckman AU5800 chemistry analyzer (Beckman Coulter, United States). TC was analyzed *via* a cholesterol oxidase enzymatic method (Beckman Coulter, United States), LDL-C and HDL-C were analyzed *via* a direct enzymatic method (Beckman Coulter, United States), and TG was analyzed *via* the GPO-PAP method (Beckman Coulter, United States). The intra-assay laboratory coefficients of variation ranged from 0.50% to 1.53%, and the between-assay coefficients of variation ranged from 1.08% to 3.55%. The diagnostic criteria for abnormal serum lipid levels were based on the Chinese Guidelines on Prevention and Treatment of Dyslipidemia in Adults (18), and were as follows: TC ≥ 6.22 mmol/L = abnormal; TG ≥ 2.26 mmol/L = abnormal; HDL-C < 1.04 mmol/L = abnormal; LDL-C ≥ 4.14 mmol/L = abnormal.

### Ovarian Stimulation

In the short-acting gonadotrophin-releasing hormone agonist (GnRH-a) (Triptorelin, Ferring AG, Germany) protocol, GnRH-a was administrated daily in the mid-luteal phase of the preceding cycle. After 14 days, serum luteinizing hormone (LH), FSH, and E2 were assessed and 150–300 IU recombinant FSH (Gonal-F, Merck Serono, Switzerland) was administered daily when FSH and LH were <5 IU/L and E2 was <50 pg/ml. GnRH-a was continued until trigger. In the subsequent GnRH-A (Cetrorelix, Merck Serono, France) protocol, 150–300 IU recombinant FSH was initiated on day 2 or day 3 of the menstrual cycle and maintained until trigger, and 0.25 mg GnRH-A was used daily when the leading follicles reached a mean diameter of 14 mm until trigger. During both protocols, if three follicles reached a mean diameter of 17 mm or two follicles reached a mean diameter of 18 mm, recombinant hCG (Ovidrel, Serono, Italy) was administered subcutaneously. Oocyte retrieval was performed 36 h after hCG injection *via* transvaginal ultrasound-guided single-lumen needle aspiration. Retrieved oocytes were inseminated *via* conventional IVF, ICSI, or IVF/ICSI (IVF 50%/ICSI 50%). Embryos were cultured in G1-plus medium (Vitrolife, Switzerland) at 37°C in an incubator with 6% carbon dioxide. Embryo transfer was performed on day 3 after fertilization in cleavage stage.

### Outcome Measures

The primary outcome was live birth. Secondary outcomes were ovarian stimulation parameters, including endometrial thickness, the number of oocytes retrieved, clinical pregnancy, and miscarriage. All women were followed to determine these reproductive outcomes. Live birth was defined as the delivery of a viable infant after 28 weeks. Clinical pregnancy was confirmed by the visualization of at least one gestational sac on ultrasound. Miscarriage was defined as spontaneous clinical pregnancy loss before 28 weeks of gestation.

### Statistical Analysis

SPSS statistics 24.0 (IBM) was used to analyze the data. Means and standard deviations, frequencies, and percentages derived from demographic and reproductive characteristics data were calculated. Patients were divided into four quartiles based on serum lipid levels. To identify possible confounders, demographic characteristics including age, BMI, duration of infertility, initial infertility diagnosis, smoker status, primary infertility, glucose, day 3 FSH level, basal E2, and AMH in the different serum lipid quartiles were compared *via* analysis of variance.

Linear regression was used to evaluate associations between endometrial thickness, the number of oocytes retrieved, and serum lipid levels before and after adjusting for possible confounders. Adjusted means with 95% confidence intervals (CIs) were calculated. Trends in endometrial thickness and the number of oocytes retrieved in the serum lipid quartiles were determined by entering the median value of each quartile and viewing them as a continuous variable in a generalized linear model. Live birth rates, clinical pregnancy rates, and miscarriage rates in serum TC, LDL-C, HDL-C, and TG quartiles were compared using chi-square tests. Logistic regression analyses were used to identify potential effects of serum lipids on pregnancy, live birth, and miscarriage before and after adjusting for possible confounders. Serum lipid levels were viewed as both continuous and categorical variables. Effects were described as odds ratios (ORs) with 95% CIs. All reported *p* values are two sided, and *p* < 0.05 was considered statistically significant.

## Results

The clinical and demographic characteristics of the 2011 women included in the study are shown in **Table 1**. The mean age was 31.0 ± 4.1 years, and the mean BMI was 21.7 ± 2.8. With regard to weight 1410 (70.1%) were normal, 199 (9.9%) were underweight, 368 (18.3%) were overweight, and 34 (1.7%) were obese. Infertility diagnosis, smoker status, duration of infertility, primary infertility, AMH, day 3 FSH, TC, LDL-C, HDL-C, TG, glucose, stimulation duration, stimulation dose, E2 level on day of hCG, endometrial thickness, type of fertilization, number of oocytes retrieved, number of two pronuclei embryos, and number of embryo transfers were also recorded. The respective incidences of abnormal TC, TG, LDL-C, and HDL-C were 1.6%, 5.2%, 2.5%, and 6.2%, respectively. To identify possible confounders, patient characteristics in the different serum lipid quartiles were compared (**Supplemental Table 1–4**). Age, BMI, infertility diagnosis, duration of infertility, AMH, day 3 FSH, basal E2, and glucose were considered potential confounders and were adjusted for in subsequent analyses.

**Table 1:** Characteristics of included women.

Characteristics	N	Mean ± SD or frequency
Age (year)	2011	31.0 ± 4.1
BMI (kg/m^2^)	2011	21.7 ± 2.8
Underweight (<18.5)	199	9.9%
Normal (18.5–23.9)	1410	70.1%
Overweight (24.0-27.9)	368	18.3%
Obese (>28.0)	34	1.7%
Infertility diagnosis		
Female	1237	61.5%
Male	305	15.2%
Unexplained	145	7.2%
Other/mixed	324	16.1%
Current smoking	23	1.1%
Duration of infertility (year)	2011	3.2 ± 2.7
Primary infertility	998	49.6%
AMH (ng/ml)	1893	3.4 ± 2.3
Day 3 FSH (IU/l)	2011	6.6 ± 2.7
Basal E2 (pg/ml)	1877	125.0 ± 64.3
TC (mmol/l)	2011	4.4 ± 0.8
Abnormal TC	32	1.6%
LDL-C (mmol/l)	2011	2.6 ± 0.7
Abnormal LDL-C	50	2.5%
HDL-C (mmol/l)	2011	1.3 ± 0.3
Abnormal HDL-C	124	6.2%
TG (mmol/l)	2011	1.1 ± 0.6
Abnormal TG	104	5.2%
Glucose (mmol/l)	1891	5.1 ± 0.6
Stimulation days (n)	2011	10.1 ± 2.3
Total gonadotropin dose (IU)	2011	2194.6 ± 810.6
E2 on day of hCG (pg/ml)	1944	2489.4 ± 1208.5
Endometrial thickness (mm)	1972	10.7 ± 2.3
Fertilization procedure		
IVF	1475	73.3%
ICSI	434	21.6%
IVF+ICSI	102	5.1%
Number of oocytes retrieved	2011	9.6 ± 4.4
Number of 2PN	2011	5.3 ± 3.3
Number of embryo transfers	2011	1.8 ± 0.4

BMI, body mass index; AMH, anti-Mullerian hormone; FSH, follicle-stimulating hormone; TC, total cholesterol; LDL-C, low-density lipoprotein cholesterol; HDL-C, high-density lipoprotein cholesterol; and TG, triglycerides; E2, estradiol; hCG, human choriogonadotropin; IVF, in vitro fertilization; ICSI, intracytoplasmic sperm injection.

Associations between serum lipid levels, endometrial thickness, and number of oocytes retrieved are shown in **Table 2**. TC, LDL-C, and TG were not significantly associated with endometrial thickness or the number of oocytes retrieved. Increasing HDL-C quartiles were associated with more oocytes retrieved 9.40 (9.21, 9.60), 9.41 (9.21, 9.62), 9.70 (9.51, 9.88), 9.87 (9.67, 10.06) after adjusting for confounders (*p* = 0.010). When lipid levels were included in analyses as continuous variables, the associations were similar to those obtained when they were included in analyses as categorical variables (**Supplemental Table 5**).

**Table 2:** Association between ovarian stimulation outcomes and serum lipids according to quartiles.

	Endometrial thickness (mm)*	P value	Number of oocytes retrieved*	P value
TC				
Q1	10.86 (10.84, 10.87)	0.458	9.55 (9.37, 9.72)	0.796
Q2	10.76 (10.74, 10.77)		9.55 (9.36, 9.74)	
Q3	10.68 (10.67, 10.70)		9.54 (9.35, 9.73)	
Q4	10.59 (10.57, 10.61)		9.76 (9.55, 9.96)	
LDL-C				
Q1	10.83 (10.81, 10.84)	0.460	9.75 (9.57, 9.93)	0.343
Q2	10.76 (10.74, 10.78)		9.63 (9.44, 9.81)	
Q3	10.69 (10.67, 10.71)		9.48 (9.30, 9.66)	
Q4	10.62 (10.61, 10.64)		9.55 (9.33, 9.76)	
HDL-C				
Q1	10.71 (10.69, 10.73)	0.327	**9.40 (9.21, 9.60)**	**0.010**
Q2	10.71 (10.69, 10.73)		**9.41 (9.21, 9.62)**	
Q3	10.72 (10.71, 10.74)		**9.70 (9.51, 9.88)**	
Q4	10.74 (10.73, 10.76)		**9.87 (9.67, 10.06)**	
TG				
Q1	10.77 (10.76, 10.79)	0.538	9.65 (9.46, 9.83)	0.835
Q2	10.75 (10.73, 10.76)		9.59 (9.40, 9.78)	
Q3	10.72 (10.71, 10.74)		9.56 (9.37, 9.75)	
Q4	10.64 (10.62, 10.66)		9.60 (9.40, 9.80)	

TC, total cholesterol; LDL-C, low-density lipoprotein cholesterol; HDL-C, high-density lipoprotein cholesterol; and TG, triglycerides; Q, quartile.

*Adjusted for age, BMI, infertility factor, duration of infertility, AMH, day 3 FSH, basal E2, glucose. Bold values indicate P value < 0.05.

**Figure 2:**
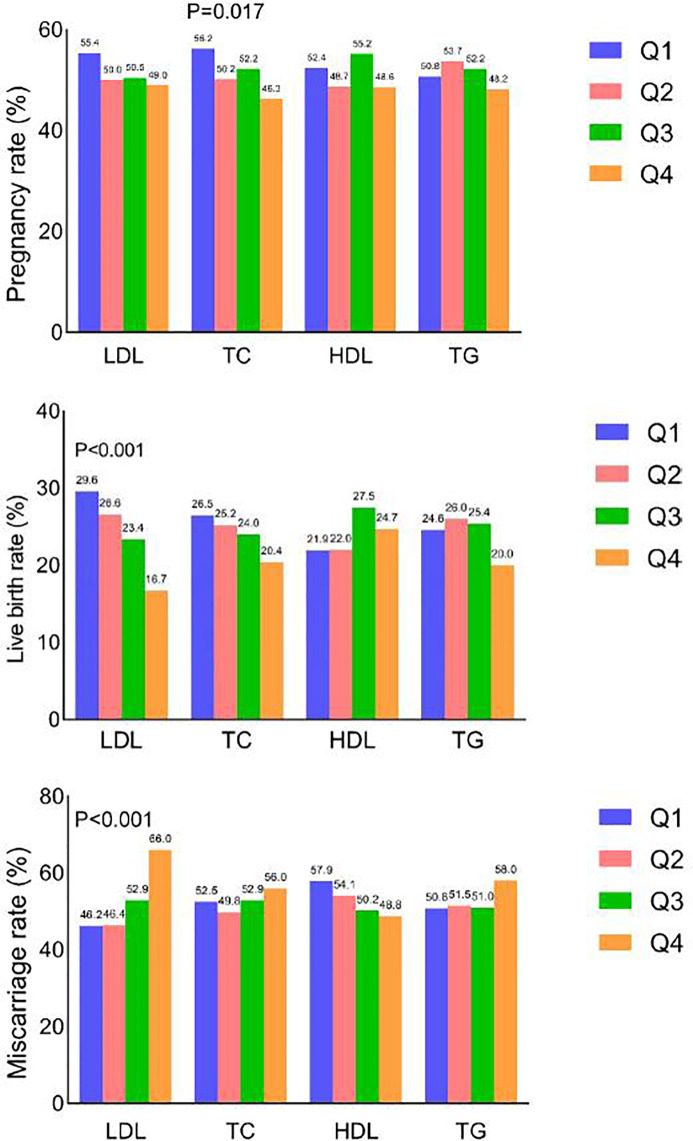
Clinical pregnancy, live birth and miscarriage rates according to quartile of each serum lipid level.

A total of 1030/2011 women (51.2%) achieved clinical pregnancy, 483 (24.0%) had live births, and 547 (53.1%) had miscarriages. Reproductive outcomes in the different serum lipid quartiles are shown in **Figure 2**. Clinical pregnancy rates in the TC quartiles differed significantly (*p* = 0.017). Live birth rates reduced as LDL-C quartile increased (*p* < 0.001). Miscarriage rates increased as LDL-C quartile increased (*p* < 0.001).

Crude and adjusted ORs of serum lipid levels for clinical pregnancy, live birth, and miscarriage compared to the highest quartile are shown in **Tables 3**, **4**, **5**. After adjusting for potential confounders, women in TC quartiles 1 (OR 1.59 [1.21–2.08]) and 3 (OR 1.36 [1.04–1.77]), LDL-C quartile 1 (OR 1.41 [1.07–1.86]), and TG quartile 2 (OR 1.39 [1.06–1.84]) were significantly more likely to achieve clinical pregnancy. After adjusting for potential confounders, women in LDL-C quartiles 1 (OR 2.22 [1.58–3.13]), 2 (OR 1.78 [1.27–2.50]), and 3 (OR 1.51 [1.07–2.13]), TC quartile 1 (OR 1.39 [1.00–1.93]), and TG quartiles 1 (OR 1.44 [1.03–2.03]), 2 (OR 1.46 [1.04–2.04]), and 3 (OR 1.44 [1.04–1.99]) were significantly more likely to have a live birth, whereas HDL-C quartile 2 (OR 0.71 [0.51–0.99]) was associated with a lower chance of live birth. After adjusting for potential confounders, women in LDL-C quartiles 1 (OR 0.44 [0.30–0.66]), 2 (OR 0.49 [0.33–0.73]), and 3 (OR 0.63 [0.43–0.94]) were less likely to have a miscarriage, whereas HDL-C quartile 1 (OR 1.60 [1.07–2.39]) was associated with an increased chance of miscarriage. When serum lipid levels were included in analyses as continuous variables, the directions of associations were similar to those obtained when they were included in analyses as categorical variables (**Supplemental Tables 6–8**).

**Table 3:** Association between pregnancy and serum lipids according to quartiles.

	Crude OR (95%CI)		Adjusted OR (95%CI) *	P value
TC				
Q1	**1.48 (1.16–1.90)**	**0.002**	**1.59 (1.21–2.08)**	**0.001**
Q2	1.17 (0.91–1.50)	0.221	1.23 (0.94–1.61)	0.125
Q3	1.26 (0.99–1.62)	0.063	**1.36 (1.04–1.77)**	**0.025**
Q4	1		1	
LDL-C				
Q1	**1.29 (1.01–1.66)**	**0.043**	**1.41 (1.07–1.86)**	**0.014**
Q2	1.04 (0.81–1.33)	0.756	1.07 (0.82–1.40)	0.615
Q3	1.06 (0.83–1.36)	0.638	1.09 (0.84–1.43)	0.510
Q4	1		1	
HDL-C				
Q1	1.16 (0.91–1.49)	0.230	1.07 (0.80–1.42)	0.665
Q2	1.00 (0.78–1.28)	0.984	0.89 (0.68–1.17)	0.413
Q3	**1.30 (1.02–1.67)**	**0.036**	1.21 (0.93–1.57)	0.167
Q4	1		1	
TG				
Q1	1.11 (0.87–1.42)	0.413	1.27 (0.96–1.69)	0.091
Q2	1.25 (0.97–1.59)	0.083	**1.39 (1.06–1.84)**	**0.019**
Q3	1.17 (0.92–1.50)	0.208	1.21 (0.92–1.58)	0.167
Q4	1		1	

TC, total cholesterol; LDL-C, low-density lipoprotein cholesterol; HDL-C, high-density lipoprotein cholesterol; and TG, triglycerides; Q, quartile.

*Adjusted for age, BMI, infertility factor, duration of infertility, AMH, day 3 FSH, basal E2, glucose. Bold values indicate P value < 0.05.

**Table 4:** Association between live birth and serum lipids according to quartiles.

	Crude OR (95%CI)		Adjusted OR (95%CI) *	P value
TC				
Q1	**1.41 (1.05–1.89)**	**0.023**	**1.39 (1.00–1.93)**	**0.047**
Q2	1.32 (0.98–1.77)	0.070	1.33 (0.96–1.84)	0.088
Q3	1.23 (0.92–1.66)	0.168	1.34 (0.97–1.86)	0.076
Q4	1		1	
LDL-C				
Q1	**2.10 (1.56–2.84)**	**<0.001**	**2.22(1.58–3.13)**	**<0.001**
Q2	**1.81 (1.34–2.46)**	**<0.001**	**1.78 (1.27–2.50)**	**<0.001**
Q3	**1.53 (1.12–2.08)**	**0.008**	**1.51 (1.07–2.13)**	**0.019**
Q4	1		1	
HDL-C				
Q1	0.85 (0.64–1.14)	0.289	0.75 (0.53–1.05)	0.093
Q2	0.86 (0.64–1.15)	0.300	**0.71 (0.51–0.99)**	**0.045**
Q3	1.16 (0.87–1.53)	0.310	1.04 (0.77–1.41)	0.800
Q4	1		1	
TG				
Q1	1.30 (0.97–1.75)	0.083	**1.44 (1.03–2.03)**	**0.036**
Q2	**1.41 (1.05–1.89)**	**0.024**	**1.46 (1.04–2.04)**	**0.028**
Q3	**1.36 (1.01–1.83)**	**0.043**	**1.44 (1.04–1.99)**	**0.028**
Q4	1		1	

TC, total cholesterol; LDL-C, low-density lipoprotein cholesterol; HDL-C, high-density lipoprotein cholesterol; and TG, triglycerides, Q, quartile.

*Adjusted for age, BMI, infertility factor, duration of infertility, AMH, day 3 FSH, basal E2, glucose. Bold values indicate P value < 0.05.

**Table 5:** Association between miscarriage and serum lipids according to quartiles.

	Crude OR (95%CI)		Adjusted OR (95%CI) *	P value
TC				
Q1	0.87 (0.61–1.23)	0.427	0.95 (0.64–1.40)	0.777
Q2	0.78 (0.55–1.12)	0.173	0.84 (0.56–1.24)	0.367
Q3	0.88 (0.62–1.26)	0.484	0.90 (0.61–1.32)	0.588
Q4	1		1	
LDL-C				
Q1	**0.44 (0.31–0.63)**	**<0.001**	**0.44 (0.30–0.66)**	**<0.001**
Q2	**0.45 (0.31–0.64)**	**<0.001**	**0.49 (0.33–0.73)**	**<0.001**
Q3	**0.58 (0.41–0.83)**	**0.003**	**0.63 (0.43–0.94)**	**0.023**
Q4	1		1	
HDL-C				
Q1	**1.44 (1.02–2.04)**	**0.041**	**1.60 (1.07–2.39)**	**0.022**
Q2	1.24 (0.87–1.76)	0.239	1.45 (0.98–2.15)	0.067
Q3	1.06 (0.75–1.49)	0.752	1.10 (0.76–1.59)	0.606
Q4	1		1	
TG				
Q1	0.75 (0.52–1.06)	0.106	0.73 (0.49–1.10)	0.129
Q2	0.77 (0.54–1.09)	0.138	0.79 (0.53–1.17)	0.232
Q3	0.75 (0.53–1.07)	0.111	0.71 (0.48–1.04)	0.079
Q4	1		1	

TC, total cholesterol; LDL-C, low-density lipoprotein cholesterol; HDL-C, high-density lipoprotein cholesterol; and TG, triglycerides; Q, quartile.

*Adjusted for age, BMI, infertility factor, duration of infertility, AMH, day 3 FSH, basal E2, glucose. Bold values indicate P value < 0.05.

## Discussion

In the current retrospective cohort study serum lipid levels were associated with treatment outcomes in women undergoing assisted reproduction. Overall, LDL-C had the strongest negative effect on reproduction outcomes including lower pregnancy rate, lower live birth rate, and higher miscarriage rate. TG and TC were also negatively associated with pregnancy and live birth rate. Conversely, higher HDL-C was associated with greater numbers of oocytes retrieved, higher live birth rates, and lower miscarriage rates.

Some previous studies have investigated relationships between lipids and embryos. In the current study, serum HDL-C was positively associated with the number of oocytes retrieved. In female reproduction, follicular fluid is the microenvironment for oocyte maturation and development. The aforementioned association may be partly explained by the anti-inflammatory and antioxidant role of HDL-C (19), which is increasingly considered to be beneficial for reproductive health (20). In the preovulatory period, follicular fluid contains only HDL which originates from blood circulation (21). HDL in follicular fluid reportedly has considerable antioxidative properties and plays an important role in the development of human oocytes (15–17, 22). In a previous study, an altered endometrial lipid profile during the peri-implantation period was associated with endometrial receptivity (10). In the current study, serum lipids were not significantly associated with endometrial thickness, which is associated with the live birth rate (23). Endometrial thickness is not the sole marker of endometrial receptivity (24), and preconception serum lipid may not completely account for local endometrial lipid profile changes during the implantation window.

The results of the current study are similar to those of some previous studies that investigated preconception lipid concentrations and reproductive outcomes in women. In a population-based prospective cohort study that included 501 couples, increased total lipid and free cholesterol concentrations as continuous variables in women were associated with a reduced fecundity OR as assessed by time to hCG-detected pregnancy (12). In another randomized controlled trial that included 1228 women with prior pregnancy loss attempting pregnancy for up to six menstrual cycles, a secondary analysis was performed to investigate associations between preconception lipid levels and fecundity, which was determined by a positive hCG test or ultrasound (13). That study used clinical cutoff points, and fecundability was reduced at all abnormal levels of TC, LDL-C, HDL-C, and TG. In the current study, significant associations between reproductive outcomes and serum lipid levels were evident when they were analyzed as both continuous variables and categorical variables. The study included patients who were young and lean, hence the low incidence of altered serum lipid profiles (ranging from 1.6%–6.2% depending on the lipid assessed). It can be speculated that in older women and obese women, among whom elevated serum lipids are more common (25, 26), associations between lipids and reproductive outcomes will be stronger. These findings further underline the role of serum lipids in reproductive outcomes in women.

The mechanisms behind associations between serum lipids and reproductive outcomes are not fully understood. Serum lipids may influence steroidogenesis. Progesterone, estrogens, androgens, and glucocorticoids are essential from embryo implantation to fetal development  (27). The oocyte is unable to produce cholesterol, and therefore relies on maternal serum lipids to provide cholesteryl esters for granulosa cell steroidogenesis. HDL-C is the main transporter of cholesterol to granulosa cells for steroidogenesis (28), which is supplied from maternal circulation. The process of steroidogenesis is gradually taken over by the placenta as it develops during the first trimester. Placental steroid synthesis is dependent on delivery from the maternal circulation. Altered maternal lipids may alter steroidogenesis and ultimately influence pregnancy outcomes. In addition, placental dysfunction is a possible cause of multiple pregnancy complications including miscarriage, preeclampsia, stillbirth, and preterm delivery (29–33). Altered lipid metabolism may affect placental lipid transport and fetal development (34). Maternal, fetal, and placental components form a unit to maintain a healthy pregnancy, and the maternal component includes serum lipids. Combined, these considerations provide plausible explanations for the independent associations between serum lipid levels and reproductive outcomes in the women undergoing assisted reproduction in the current study.

Serum lipids are routinely monitored in clinical settings as indicators of cardiovascular system health. The results of the current study suggest that serum lipids are also predictors of outcomes in women undergoing assisted reproduction. The high prevalence of lipid abnormalities in modern populations facilitates an opportunity to evaluate lipid profiles in an effort to predict reproductive outcomes in these populations. Abnormal serum lipid levels are usually modified by increased physical activity, diet modification, or statins (35–37). The current study suggests that there may be benefits to improving serum lipid levels before assisted reproduction treatment. Future clinical studies should focus on whether pretreatment to optimize lipid levels *via* interventions such as physical activity, diet modification, or statins can improve reproductive outcomes.

The major strength of the current study was the large sample size. The observed associations remained significant after adjustment for a number of potential confounders. Despite efforts to eliminate sources of bias in the study, it also had several limitations. One is the inevitable bias due to its retrospective nature. Despite the large sample size, the adjustments made, and the multivariable regression analysis conducted, the presence of confounding bias cannot be excluded. In addition, having in mind that our studied population was heterogeneous, more research is needed in specific patient subpopulations where one might expect that altered lipid profiles may affect treatment outcomes, such as polycystic ovary syndrome patients. As only a single measure of preconception serum lipid levels was available, it was not possible to investigate associations between lipid levels in sequential cycles, and due to the study design it was also not possible to investigate whether serum lipids were related to the cumulative live birth rate. Lastly, data on time elapsed from the measurement of serum lipids and the initiation of IVF were unavailable, and future studies could investigate this.

The results of the current study constitute evidence on the role of serum lipids in treatment outcomes in women undergoing assisted reproduction. Among women who aim to achieve good outcomes from assisted reproduction, a lipid panel test may serve as an early, simple, inexpensive, and modifiable predictor. Further studies are needed to expand our understanding of whether interventions aimed at improving serum lipid profiles can optimize assisted pregnancy outcomes.

## Data Availability Statement

The original contributions presented in the study are included in the article/**Supplementary Material**. Further inquiries can be directed to the corresponding authors.

## Ethics Statement

The studies involving human participants were reviewed and approved by Hospital Ethics Committee, Women’s Hospital, School of Medicine, Zhejiang University. Written informed consent for participation was not required for this study in accordance with the national legislation and the institutional requirements.

## Author Contributions

X-KW and JX designed the study and critically revised the manuscript. W-YC and XL performed data analysis. W-YC, XL, EC, HL, and KF collected data. W-YC and XL drafted the manuscript. All authors contributed to the article and approved the submitted version.

## Conflict of Interest

The authors declare that the research was conducted in the absence of any commercial or financial relationships that could be construed as a potential conflict of interest.

## References

[1] Mascarenhas MN Flaxman SR Boerma T Vanderpoel S Stevens GA. National, regional, and global trends in infertility prevalence since 1990: a systematic analysis of 277 health surveys. PLoS Med (2012) 9(12):e1001356. 10.1371/journal.pmed.1001356 23271957 PMC3525527

[2] Wu AK Elliott P Katz PP Smith JF. Time costs of fertility care: the hidden hardship of building a family. Fertil Steril (2013) 99(7):2025–30. 10.1016/j.fertnstert.2013.01.145 PMC3736984 23454007

[3] Rooney KL Domar AD. The relationship between stress and infertility. Dialog Clin Neurosci (2018) 20(1):41–7. 10.31887/DCNS.2018.20.1/klrooney PMC6016043 29946210

[4] Kushnir VA Barad DH Albertini DF Darmon SK Gleicher N. Systematic review of worldwide trends in assisted reproductive technology 2004-2013. Reprod Biol Endocrinol (2017) 15(1):6–. 10.1186/s12958-016-0225-2 PMC5223447 28069012

[5] NCD Risk Factor Collaboration (NCD-RisC). Worldwide trends in body-mass index, underweight, overweight, and obesity from 1975 to 2016: a pooled analysis of 2416 population-based measurement studies in 128·9 million children, adolescents, and adults. Lancet (2017) 390(10113):2627–42. 10.1016/S0140-6736(17)32129-3 PMC5735219 29029897

[6] Tóth PP Potter D Ming EE. Prevalence of lipid abnormalities in the United States: the National Health and Nutrition Examination Survey 2003-2006. J Clin Lipidol (2012) 6(4):325–30. 10.1016/j.jacl.2012.05.002 22836069

[7] Huang Y Gao L Xie X Tan SC. Epidemiology of dyslipidemia in Chinese adults: meta-analysis of prevalence, awareness, treatment, and control. Popul Health Metr (2014) 12(1):28. 10.1186/s12963-014-0028-7 25371655 PMC4219092

[8] Miller WL. Molecular biology of steroid hormone synthesis. Endocr Rev (1988) 9(3):295–318. 10.1210/edrv-9-3-295 3061784

[9] Huang Q Liu Y Yang Z Xie Y Mo Z. The Effects of Cholesterol Metabolism on Follicular Development and Ovarian Function. Curr Mol Med (2019) 19(10):719–30. 10.2174/1566524019666190916155004 31526349

[10] Li J Gao Y Guan L Zhang H Chen P Gong X. Lipid Profiling of Peri-implantation Endometrium in Patients With Premature Progesterone Rise in the Late Follicular Phase. J Clin Endocrinol Metab (2019) 104(11):5555–65. 10.1210/jc.2019-00793 31390011

[11] Brosens I Brosens JJ Muter J Benagiano G. Acute atherosis and diffuse lipid infiltration of the placental bed: A review of historical lipid studies. Placenta (2020) 97:36–41. 10.1016/j.placenta.2020.06.012 32792060

[12] Schisterman EF Mumford SL Browne RW Barr DB Chen Z Louis GMB. Lipid concentrations and couple fecundity: the LIFE study. J Clin Endocrinol Metab (2014) 99(8):2786–94. 10.1210/jc.2013-3936 PMC4121020 24846535

[13] Pugh SJ Schisterman EF Browne RW Lynch AM Mumford SL Perkins NJ. Preconception maternal lipoprotein levels in relation to fecundability. Hum Reprod (2017) 32(5):1055–63. 10.1093/humrep/dex052 PMC6075456 28333301

[14] Browne RW Shelly WB Bloom MS Ocque AJ Sandler JR Huddleston HG. Distributions of high-density lipoprotein particle components in human follicular fluid and sera and their associations with embryo morphology parameters during IVF. Hum Reprod (2008) 23(8):1884–94. 10.1093/humrep/den183 18487218

[15] Browne RW Bloom MS Shelly WB Ocque AJ Huddleston HG Fujimoto VY. Follicular fluid high density lipoprotein-associated micronutrient levels are associated with embryo fragmentation during IVF. J Assist Reprod Genet (2009) 26(11-12):557–60. 10.1007/s10815-009-9367-x PMC2799562 19921421

[16] Nagy RA van Montfoort APA Groen H Homminga I Andrei D Mistry RH. Anti-oxidative function of follicular fluid HDL and outcomes of modified natural cycle-IVF. Sci Rep (2019) 9(1):12817. 10.1038/s41598-019-49091-3 31492916 PMC6731220

[17] Bacchetti T Morresi C Vignini A Tiano L Orlando P Montik N. HDL functionality in follicular fluid in normal-weight and obese women undergoing assisted reproductive treatment. J Assist Reprod Genet (2019) 36(8):1657–64. 10.1007/s10815-019-01523-9 PMC6708027 31338723

[18] Joint Committee for Developing Chinese guidelines on Prevention and Treatment of Dyslipidemia in Adults. [Chinese guidelines on prevention and treatment of dyslipidemia in adults]. Zhonghua Xin Xue Guan Bing Za Zhi (2007) 35(5):390–419. 10.3760/j.issn:0253-3758.2007.05.003 17711682

[19] Soran H Schofield JD Durrington PN. Antioxidant properties of HDL. Front Pharmacol (2015) 6:222. 10.3389/fphar.2015.00222 26528181 PMC4607861

[20] Fujimoto VY Kane JP Ishida BY Bloom MS Browne RW. High-density lipoprotein metabolism and the human embryo. Hum Reprod Update (2010) 16(1):20–38. 10.1093/humupd/dmp029 19700490

[21] Jaspard B Fournier N Vieitez G Atger V Barbaras R Vieu C. Structural and functional comparison of HDL from homologous human plasma and follicular fluid. A model for extravascular fluid. Arterioscler Thromb Vasc Biol (1997) 17(8):1605–13. 10.1161/01.ATV.17.8.1605 9301642

[22] Kim K Bloom MS Browne RW Bell EM Yucel RM Fujimoto VY. Associations between follicular fluid high density lipoprotein particle components and embryo quality among in vitro fertilization patients. J Assist Reprod Genet (2017) 34(1):1–10. 10.1007/s10815-016-0826-x PMC5237431 27900613

[23] Simeonov M Sapir O Lande Y Ben-Haroush A Oron G Shlush E. The entire range of trigger-day endometrial thickness in fresh IVF cycles is independently correlated with live birth rate. Reprod BioMed Online (2020) 41(2):239–47. 10.1016/j.rbmo.2020.04.008 32532669

[24] Casper RF. Frozen embryo transfer: evidence-based markers for successful endometrial preparation. Fertil Steril (2020) 113(2):248–51. 10.1016/j.fertnstert.2019.12.008 32106971

[25] Pan L Yang Z Wu Y Yin R-X Liao Y Wang J. The prevalence, awareness, treatment and control of dyslipidemia among adults in China. Atherosclerosis (2016) 248:2–9. 10.1016/j.atherosclerosis.2016.02.006 26978581

[26] Zhang L Zhang W-H Zhang L Wang P-Y. Prevalence of overweight/obesity and its associations with hypertension, diabetes, dyslipidemia, and metabolic syndrome: a survey in the suburban area of Beijing, 2007. Obes Facts (2011) 4(4):284–9. 10.1159/000331014 PMC6444797 21921651

[27] Morel Y Roucher F Plotton I Goursaud C Tardy V Mallet D. Evolution of steroids during pregnancy: Maternal, placental and fetal synthesis. Ann Endocrinol (Paris) (2016) 77(2):82–9. 10.1016/j.ando.2016.04.023 27155772

[28] Azhar S Tsai L Medicherla S Chandrasekher Y Giudice L Reaven E. Human granulosa cells use high density lipoprotein cholesterol for steroidogenesis. J Clin Endocrinol Metab (1998) 83(3):983–91. 10.1210/jcem.83.3.4662 9506760

[29] Gun BD Numanoglu G Ozdamar SO. The comparison of vessels in elective and spontaneous abortion decidua in first trimester pregnancies: importance of vascular changes in early pregnancy losses. Acta Obstet Gynecol Scand (2006) 85(4):402–6. 10.1080/00016340500501731 16612700

[30] Lambropoulou M Tamiolakis D Venizelos J Liberis V Galazios G Tsikouras P. Imbalance of mononuclear cell infiltrates in the placental tissue from foetuses after spontaneous abortion versus therapeutic termination from 8th to 12th weeks of gestational age. Clin Exp Med (2006) 6(4):171–6. 10.1007/s10238-006-0111-x 17191109

[31] Guzin K Tomruk S Tuncay YA Naki M Sezginsoy S Zemheri E. The relation of increased uterine artery blood flow resistance and impaired trophoblast invasion in pre-eclamptic pregnancies. Arch Gynecol Obstet (2005) 272(4):283–8. 10.1007/s00404-005-0005-2 16007505

[32] Romero R Kusanovic JP Chaiworapongsa T Hassan SS. Placental bed disorders in preterm labor, preterm PROM, spontaneous abortion and abruptio placentae. Best Pract Res Clin Obstet Gynaecol (2011) 25(3):313–27. 10.1016/j.bpobgyn.2011.02.006 PMC3092823 21388889

[33] Wijaya JC Khanabdali R Georgiou HM Kokkinos MI James PF Brennecke SP. Functional changes in decidual mesenchymal stem/stromal cells are associated with spontaneous onset of labour. Mol Hum Reprod (2020) 26(8):636–51. 10.1093/molehr/gaaa045 32609359

[34] Delhaes F Giza SA Koreman T Eastabrook G McKenzie CA Bedell S. Altered maternal and placental lipid metabolism and fetal fat development in obesity: Current knowledge and advances in non-invasive assessment. Placenta (2018) 69:118–24. 10.1016/j.placenta.2018.05.011 29907450

[35] Hasan B Nayfeh T Alzuabi M Wang Z Kuchkuntla AR Prokop LJ. Weight Loss and Serum Lipids in Overweight and Obese Adults: A Systematic Review and Meta-Analysis. J Clin Endocrinol Metab (2020) 105(12):3695–703. 10.1210/clinem/dgaa673 32954416

[36] Ge L Sadeghirad B Ball GDC da Costa BR Hitchcock CL Svendrovski A. Comparison of dietary macronutrient patterns of 14 popular named dietary programmes for weight and cardiovascular risk factor reduction in adults: systematic review and network meta-analysis of randomised trials. BMJ (2020) 369:m696. 10.1136/bmj.m696 32238384 PMC7190064

[37] Delahoy PJ Magliano DJ Webb K Grobler M Liew D. The relationship between reduction in low-density lipoprotein cholesterol by statins and reduction in risk of cardiovascular outcomes: an updated meta-analysis. Clin Ther (2009) 31(2):236–44. 10.1016/j.clinthera.2009.02.017 19302897

