# Supplementary material for: Serum Lipid Levels and Treatment Outcomes in Women Undergoing Assisted Reproduction: A Retrospective Cohort Study
Source: Front Endocrinol (Lausanne). 2021 Mar 8;12:633766. doi: 10.3389/fendo.2021.633766 (PMC7982671; doi:10.3389/fendo.2021.633766)
Supplement: Supplementary file 1 [file Table_1.docx]

| Supplemental table 1 Patients characteristics across quartiles of LDL-C | | | | | |
| --- | --- | --- | --- | --- | --- |
| Characteristics | Q1 | Q2 | Q3 | Q4 | P value |
| Age (year) | 30.3 ± 3.9 | 30.8 ± 4.1 | 31.2 ± 3.8 | 31.6 ± 4.5 | <0.001 |
| BMI (kg/m^2^) | 21.0 ± 2.4 | 21.4 ± 2.9 | 21.8 ± 2.6 | 22.5 ± 2.9 | <0.001 |
| <18.5 | 71 | 56 | 44 | 28 | <0.001 |
| 18.5–23.9 | 368 | 353 | 353 | 336 |  |
| 24.0-27.9 | 58 | 80 | 102 | 128 |  |
| >28.0 | 3 | 7 | 6 | 18 |  |
| Infertility diagnosis |  |  |  |  |  |
| Female | 299 | 295 | 340 | 303 | 0.055 |
| Male | 75 | 69 | 70 | 91 |  |
| Unexplained | 38 | 36 | 34 | 37 |  |
| Other/mixed | 88 | 96 | 61 | 79 |  |
| Current smoking | 6 | 7 | 6 | 4 | 0.821 |
| Duration of infertility (year) | 3.1 ± 2.6 | 3.2 ± 2.9 | 3.1 ± 2.4 | 3.3 ± 2.9 | 0.695 |
| Primary infertility | 259 | 248 | 234 | 257 | 0.732 |
| AMH (ng/ml) | 3.2 ± 2.1 | 3.3 ± 2.3 | 3.3 ± 2.3 | 3.6 ± 2.6 | 0.066 |
| Day 3 FSH (IU/l) | 6.5 ± 2.6 | 6.7 ± 2.8 | 6.6 ± 2.6 | 6.4 ± 2.8 | 0.243 |
| Basal E2 | 131.6 ± 66.8 | 126.1 ± 64.4 | 123.5 ± 63.2 | 118.7 ± 62.2 | 0.021 |
| TC (mmol/l) | 3.7 ± 0.6 | 4.2 ± 0.4 | 4.6 ± 0.5 | 5.3 ± 0.7 | <0.001 |
| LDL-C (mmol/l) | 1.8 ± 0.3 | 2.3 ± 0.1 | 2.8 ± 0.1 | 3.5 ± 0.5 | 0.001 |
| HDL-C (mmol/l) | 1.4 ± 0.3 | 1.4 ± 0.3 | 1.3 ± 0.3 | 1.3 ± 0.3 | <0.001 |
| TG (mmol/l) | 1.0 ± 0.6 | 1.0 ± 0.6 | 1.1 ± 0.7 | 1.3 ± 0.6 | <0.001 |
| Glucose (mmol/l) | 5.1 ± 0.4 | 5.1 ± 0.5 | 5.2 ± 0.7 | 5.2 ± 0.6 | <0.001 |
| Abbreviations: BMI (body mass index), AMH (anti-Mullerian hormone), FSH (follicle-stimulating hormone), E2 (estradiol), TC (total cholesterol), LDL-C (low-density lipoprotein cholesterol), HDL-C (high-density lipoprotein cholesterol) and TG (triglycerides), Q (quartile) | | | | | |

| Supplemental table 2 Patients characteristics across quartiles of TC | | | | | |
| --- | --- | --- | --- | --- | --- |
| Characteristics | Q1 | Q2 | Q3 | Q4 | P value |
| Age (year) | 30.2 ± 4.1 | 30.7 ± 3.9 | 31.4 ± 4.0 | 31.6 ± 4.3 | <0.001 |
| BMI (kg/m^2^) | 21.5 ± 2.6 | 21.5 ± 2.8 | 21.6 ± 2.9 | 22.1 ± 2.9 | 0.005 |
| <18.5 | 58 | 50 | 46 | 45 | 0.092 |
| 18.5–23.9 | 361 | 354 | 361 | 334 |  |
| 24.0-27.9 | 75 | 91 | 88 | 114 |  |
| >28.0 | 8 | 5 | 9 | 12 |  |
| Infertility diagnosis |  |  |  |  |  |
| Female | 290 | 312 | 324 | 311 | 0.116 |
| Male | 80 | 73 | 70 | 82 |  |
| Unexplained | 29 | 37 | 42 | 37 |  |
| Other/mixed | 103 | 78 | 68 | 75 |  |
| Current smoking | 7 | 5 | 8 | 3 | 0.458 |
| Duration of infertility (year) | 3.2 ± 2.8 | 3.2 ± 2.5 | 3.2 ± 2.8 | 3.1 ± 2.6 | 0.895 |
| Primary infertility | 249 | 266 | 228 | 255 | 0.286 |
| AMH (ng/ml) | 3.2 ± 2.1 | 3.3 ± 2.3 | 3.4 ± 2.3 | 3.5 ± 2.6 | 0.162 |
| Day 3 FSH (IU/l) | 6.4 ± 2.6 | 6.7 ± 2.8 | 6.6 ± 2.7 | 6.5 ± 2.8 | 0.279 |
| Basal E2 | 125.9 ± 59.0 | 127.8 ± 68.8 | 121.7 ± 62.8 | 124.7 ± 66.2 | 0.518 |
| Total cholesterol (mmol/l) | 3.5 ± 0.4 | 4.1 ± 0.1 | 4.6 ± 0.1 | 5.4 ± 0.6 | <0.001 |
| HDL-cholesterol (mmol/l) | 1.2 ± 0.3 | 1.3 ± 0.3 | 1.4 ± 0.3 | 1.4 ± 0.3 | <0.001 |
| Triglycerides (mmol/l) | 1.0 ± 0.6 | 1.0 ± 0.6 | 1.1 ± 0.7 | 1.3 ± 0.7 | <0.001 |
| LDL-cholesterol (mmol/l) | 1.9 ± 0.4 | 2.4 ± 0.3 | 2.8 ± 0.4 | 3.4 ± 0.6 | <0.001 |
| Glucose (mmol/l) | 5.1 ± 0.4 | 5.1 ± 0.5 | 5.2 ± 0.5 | 5.2 ± 0.8 | <0.001 |
| Abbreviations: BMI (body mass index), AMH (anti-Mullerian hormone), FSH (follicle-stimulating hormone), E2 (estradiol), TC (total cholesterol), LDL-C (low-density lipoprotein cholesterol), HDL-C (high-density lipoprotein cholesterol) and TG (triglycerides), Q (quartile) | | | | | |

| Supplemental table 3 Patients characteristics across quartiles of HDL | | | | | |
| --- | --- | --- | --- | --- | --- |
| Characteristics | Q1 | Q2 | Q3 | Q4 | P value |
| Age (year) | 31.0 ± 4.4 | 31.4 ± 4.1 | 30.6 ± 4.0 | 30.9 ± 4.0 | 0.031 |
| BMI (kg/m^2^) | 23.2 ± 2.5 | 22.1 ± 2.9 | 21.0 ± 2.2 | 20.5 ± 2.6 | <0.001 |
| <18.5 | 16 | 29 | 53 | 101 | <0.001 |
| 18.5–23.9 | 299 | 349 | 394 | 368 |  |
| 24.0-27.9 | 166 | 112 | 48 | 42 |  |
| >28.0 | 17 | 11 | 3 | 3 |  |
| Infertility diagnosis |  |  |  |  |  |
| Female | 302 | 327 | 313 | 295 | 0.006 |
| Male | 93 | 62 | 70 | 80 |  |
| Unexplained | 24 | 29 | 46 | 46 |  |
| Other/mixed | 79 | 83 | 69 | 93 |  |
| Current smoking | 4 | 9 | 7 | 3 | 0.248 |
| Duration of infertility (year) | 3.2 ± 2.9 | 3.4 ± 2.8 | 3.0 ± 2.4 | 3.0 ± 2.6 | 0.047 |
| Primary infertility | 242 | 254 | 248 | 254 | 0.959 |
| AMH (ng/ml) | 3.4 ± 2.5 | 3.3 ± 2.3 | 3.4 ± 2.3 | 3.3 ± 2.2 | 0.772 |
| Day 3 FSH (IU/l) | 6.1 ± 2.5 | 6.5 ± 3.0 | 6.7 ± 2.5 | 7.0 ± 2.8 | <0.001 |
| Basal E2 | 113.6 ± 58.2 | 125.5 ± 61.8 | 127.4 ± 64.9 | 133.0 ± 69.9 | <0.001 |
| Total cholesterol (mmol/l) | 4.2 ± 0.8 | 4.3 ± 0.8 | 4.4 ± 0.8 | 4.7 ± 0.8 | <0.001 |
| HDL-cholesterol (mmol/l) | 1.0 ± 0.1 | 1.2 ± 0.1 | 1.4 ± 0.1 | 1.7 ± 0.2 | <0.001 |
| Triglycerides (mmol/l) | 1.5 ± 0.9 | 1.1 ± 0.6 | 0.9 ± 0.4 | 0.8 ± 0.4 | <0.001 |
| LDL-cholesterol (mmol/l) | 2.7 ± 0.7 | 2.6 ± 0.7 | 2.6 ± 0.7 | 2.5 ± 0.7 | 0.015 |
| Glucose (mmol/l) | 5.2 ± 0.7 | 5.2 ± 0.6 | 5.1 ± 0.5 | 5.1 ± 0.5 | 0.013 |
| Abbreviations: BMI (body mass index), AMH (anti-Mullerian hormone), FSH (follicle-stimulating hormone), E2 (estradiol), TC (total cholesterol), LDL-C (low-density lipoprotein cholesterol), HDL-C (high-density lipoprotein cholesterol) and TG (triglycerides), Q (quartile) | | | | | |

| Supplemental table 4 Patients characteristics across quartiles of TG | | | | | |
| --- | --- | --- | --- | --- | --- |
| Characteristics | Q1 | Q2 | Q3 | Q4 | P value |
| Age (year) | 30.5 ± 3.9 | 30.8 ± 4.0 | 31.3 ± 4.2 | 31.3 ± 4.3 | 0.003 |
| BMI (kg/m^2^) | 20.6 ± 2.4 | 21.1 ± 2.5 | 22.2 ± 2.7 | 22.9 ± 2.8 | <0.001 |
| <18.5 | 79 | 69 | 33 | 18 | <0.001 |
| 18.5–23.9 | 378 | 375 | 341 | 316 |  |
| 24.0-27.9 | 41 | 57 | 119 | 151 |  |
| >28.0 | 2 | 2 | 11 | 19 |  |
| Infertility diagnosis |  |  |  |  |  |
| Female | 300 | 302 | 314 | 321 | 0.936 |
| Male | 74 | 83 | 74 | 74 |  |
| Unexplained | 41 | 38 | 33 | 33 |  |
| Other/mixed | 85 | 80 | 83 | 76 |  |
| Current smoking | 3 | 5 | 9 | 6 | 0.354 |
| Duration of infertility (year) | 3.1 ± 2.5 | 3.1 ± 2.6 | 3.2 ± 2.8 | 3.3 ± 2.8 | 0.604 |
| Primary infertility | 260 | 247 | 240 | 251 | 0.734 |
| AMH (ng/ml) | 3.2 ± 2.2 | 3.2 ± 2.2 | 3.4 ± 2.3 | 3.5 ± 2.6 | 0.149 |
| Day 3 FSH (IU/l) | 6.9 ± 2.7 | 6.7 ± 2.6 | 6.6 ± 3.0 | 6.1 ± 2.6 | <0.001 |
| Basal E2 | 131.5 ± 66.3 | 126.1 ± 61.7 | 123.3 ± 62.8 | 119.0 ± 65.8 | 0.024 |
| Total cholesterol (mmol/l) | 4.2 ± 0.7 | 4.4 ± 0.7 | 4.5 ± 0.8 | 4.6 ± 0.9 | <0.001 |
| HDL-cholesterol (mmol/l) | 1.5 ± 0.3 | 1.4 ± 0.3 | 1.3 ± 0.3 | 1.2 ± 0.3 | <0.001 |
| Triglycerides (mmol/l) | 0.6 ± 0.1 | 0.8 ± 0.1 | 1.1 ± 0.1 | 1.9 ± 0.8 | <0.001 |
| LDL-cholesterol (mmol/l) | 2.4 ± 0.6 | 2.5 ± 0.7 | 2.7 ± 0.7 | 2.8 ± 0.7 | <0.001 |
| Glucose (mmol/l) | 5.0 ± 0.5 | 5.1 ± 0.4 | 5.2 ± 0.7 | 5.3 ± 0.7 | <0.001 |
| Abbreviations: BMI (body mass index), AMH (anti-Mullerian hormone), FSH (follicle-stimulating hormone), E2 (estradiol), TC (total cholesterol), LDL-C (low-density lipoprotein cholesterol), HDL-C (high-density lipoprotein cholesterol) and TG (triglycerides), Q (quartile) | | | | | |

| Supplemental table 5 Association between ovarian stimulation outcomes and serum lipids as continuous variable | | | | |
| --- | --- | --- | --- | --- |
|  | Endometrial thickness (mm)* | P value | Number of oocytes retrieved* | P value |
| TC | -0.12 (-0.26, 0.02) | 0.091 | 0.11 (-0.13, 0.34) | 0.370 |
| LDL-C | -0.12 (-0.28, 0.05) | 0.168 | -0.17 (-0.44, 0.11) | 0.235 |
| HDL-C | 0.10 (-0.31, 0.50) | 0.642 | **0.99 (0.31, 1.67)** | **0.005** |
| TG | -0.11 (-0.28, 0.07) | 0.219 | -0.21 (-0.51, 0.08) | 0.159 |
| Abbreviations: TC (total cholesterol), LDL-C (low-density lipoprotein cholesterol), HDL-C (high-density lipoprotein cholesterol) and TG (triglycerides)  *Adjusted for age, BMI, infertility factor, duration of infertility, AMH, day 3 FSH, basal E2, glucose | | | | |

| Supplemental table 6 Association between pregnancy and serum lipids as continuous variable | | | | |
| --- | --- | --- | --- | --- |
|  | Crude OR (95%CI) | P value | Adjusted OR (95%CI) * | P value |
| TC | **0.86 (0.77-0.96)** | **0.008** | **0.85 (0.75-0.96)** | **0.007** |
| LDL-C | **0.87 (0.77-0.96)** | **0.028** | **0.83 (0.72-0.95)** | **0.008** |
| HDL-C | 0.86 (0.64-1.16) | 0.332 | 0.95 (0.67-1.35) | 0.792 |
| TG | 0.96 (0.84-1.10) | 0.529 | 0.91 (0.78-1.06) | 0.218 |
| Abbreviations: TC (total cholesterol), LDL-C (low-density lipoprotein cholesterol), HDL-C (high-density lipoprotein cholesterol) and TG (triglycerides)  *Adjusted for age, BMI, infertility factor, duration of infertility, AMH, day 3 FSH, basal E2, glucose | | | | |

| Supplemental table 7 Association between live birth and serum lipids as continuous variable | | | | |
| --- | --- | --- | --- | --- |
|  | Crude OR (95%CI) | P value | Adjusted OR (95%CI) * | P value |
| TC | **0.84 (0.74-0.96)** | **0.009** | **0.85 (0.74-0.98)** | **0.026** |
| LDL-C | **0.65 (0.56-0.76)** | **<0.001** | **0.62 (0.52-0.74)** | **<0.001** |
| HDL-C | 1.30 (0.92-1.83) | 0.142 | **1.58 (1.05-2.37)** | **0.027** |
| TG | 0.90 (0.76-1.07) | 0.228 | 0.87 (0.72-1.06) | 0.172 |
| Abbreviations: TC (total cholesterol), LDL-C (low-density lipoprotein cholesterol), HDL-C (high-density lipoprotein cholesterol) and TG (triglycerides)  *Adjusted for age, BMI, infertility factor, duration of infertility, AMH, day 3 FSH, basal E2, glucose | | | | |

| Supplemental table 8 Association between miscarriage and serum lipids as continuous variable | | | | |
| --- | --- | --- | --- | --- |
|  | Crude OR (95%CI) | P value | Adjusted OR (95%CI) * | P value |
| TC | 1.12 (0.96-1.31) | 0.158 | 1.09 (0.92-1.29) | 0.325 |
| LDL-C | **1.61 (1.33-1.94)** | **<0.001** | **1.64 (1.32-2.02)** | **<0.001** |
| HDL-C | **0.60 (0.39-0.91)** | **0.018** | **0.49 (0.30-0.81)** | **0.005** |
| TG | 1.11 (0.92-1.34) | 0.293 | 1.10 (0.89-1.37) | 0.379 |
| Abbreviations: TC (total cholesterol), LDL-C (low-density lipoprotein cholesterol), HDL-C (high-density lipoprotein cholesterol) and TG (triglycerides)  *Adjusted for age, BMI, infertility factor, duration of infertility, AMH, day 3 FSH, basal E2, glucose | | | | |
